# Within and Between Subject Spectral Fingerprints of EEG-Microstate Parameters

**DOI:** 10.1007/s10548-022-00896-y

**Published:** 2022-04-12

**Authors:** Johannes Zulliger, Laura Diaz Hernandez, Thomas Koenig

**Affiliations:** Translational Research Center, University Hospital of Psychiatry, University of Bern, Bern, Switzerland

**Keywords:** Microstates, Spectral EEG, Covariance, Alpha

## Abstract

Early reports have claimed that EEG microstate features (e.g. their mean duration or percent of time covered) are largely independent from EEG spectra. This has meanwhile been questioned for conceptual and empirical reasons, but so far, EEG spectral power map correlates of microstate features have not been reported. We present the results of such analyses, conducted both within and between subjects, and report patterns of systematic changes in local EEG spectral amplitude associated with the mean duration, frequency of occurrence and relative contribution of particular microstate classes. The combination of EEG microstate analysis with spectral analysis may therefore be helpful to come to a deeper understanding of local patterns of activation and inhibition associated with particular microstate classes.

## Introduction

Since the beginning of microstate research, possible links between EEG spectral power and microstate features have been discussed (Wackermann et al. 1993; Koenig et al. 2002; Britz et al. 2010; Milz et al. 2017; Croce et al. 2020). Early studies reported no (Wackermann et al. 1993) or weak correlations (Koenig et al. 2002; Britz et al. 2010) between microstates and spectral power, but had only computed these correlations between individual power spectra averaged over all electrodes and the entire analysis time, and individual mean microstate features over time. In more recent studies, microstate features were found to differ within subjects between individual EEG epochs that were classified by their amount of occipital alpha power (Croce et al. 2020)., and it was reported that periods assigned to different microstate classes had locally different EEG spectral energy in particular frequency bands (Javed et al. 2019). In addition Michel and Koenig (2018) linked microstates to the hypothesis that communication among brain regions may be gated through coherence among slow oscillations of local cortical excitability [Communication through coherence (CTC) hypothesis (Fries 2005)], which would predict that transient changes in microstate features covary with local and transient changes in excitability observable in EEG spectral power.

However, to our knowledge, there is yet no study that directly correlated the spontaneous variance of EEG microstate features with the local variance of EEG spectra. We therefore aimed to extract spectral ‘fingerprints’ of EEG microstates by using their natural variance over time and subjects as regressor on EEG spectra. Importantly, we conducted these analyses both using the within-subject variance of the EEG, (considered as state marker), and the between subject variance of the EEG (typically considered to be a trait marker).

## Methods

**Fig. 1:**
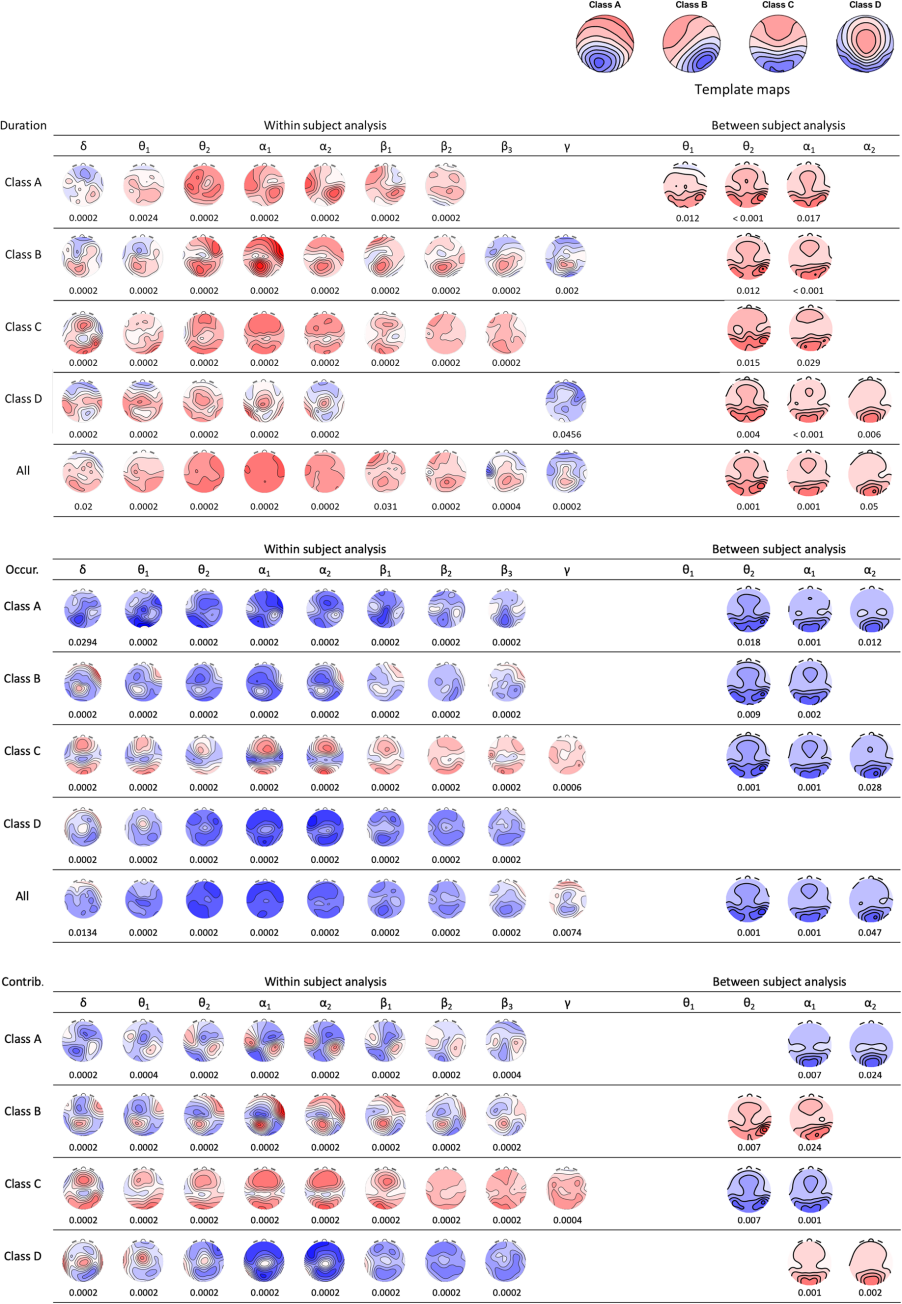
Within (left columns) and between (right columns) spectral covariates of the EEG microstate features duration (upper rows), contribution (middle rows), and occurrence (lower rows) as a function of frequency bands. Within subject covariance maps are in steps of 1 t, indicated p-values were obtained using TCTs, between-subject covariance maps have arbitrary units and were tested using TANCOVAs

The EEG employed here has been collected as part of another study (Diaz Hernandez et al. 2016) and was recorded during an initial 4 min eyes-closed resting period in 20 participants (10F/10M, mean age 24.8 ± 3.61 years) in 32 channels. The artefact edited individual EEG recordings were recomputed to average reference and partitioned into 2 s epochs. After applying a 2−20 Hz band-pass filter, 4 microstate templates previously obtained in the data (Diaz Hernandez et al. 2016, and Fig. 1) were applied to the EEG, and the EEG microstate duration, occurrence, and percent time covered were class-wise computed in each epoch. This yielded a total of 14 time-varying features (3 features × 4 classes + 1 across microstate class mean duration and 1 mean occurrence across classes). In addition, epochs were FFT transformed and epoch-wise varying absolute spectral amplitude maps were computed in nine frequency bands [Delta (1−3.5 Hz), lower (3.5−6.5 Hz) and upper (6.5−8.5 Hz) theta, lower (8.5−10.5 Hz) and upper (10.5−13 Hz) alpha, lower (13−18.5 Hz), middle (18.5−21 Hz) and upper (21−30 Hz) beta and lower gamma (30−46 Hz)]. For the between subject analyses, microstate features and spectral amplitude maps were individually averaged across epochs. There were thus 14 microstate features and 9 frequency bands, and thus in total 126 combinations among them to be correlated with each other.

### Microstate Spectral Correlates Within Subjects

For the within subject analysis, in each subject, each time-varying microstate feature was used as a regressor for the time-varying spectral amplitudes maps, yielding a map of spectral regression coefficients (covariance map) for each microstate feature, frequency band and subject. Topographic consistency tests (TCT, Koenig and Melie-García 2010) were then used to test if the obtained covariance maps were consistent between subjects. When the TCT was significant (p < 0.05), one-sample t-maps were computed across individual covariance-maps. In addition, to test whether the obtained covariance maps systematically varied by frequency band and/or microstate class, we computed TANOVAs with frequency band and microstate class as within factors. Finally, we computed and tabulated the shared spatial variance of the obtained covariance maps with their corresponding rectified microstate template maps. (Rectifying the template maps takes into account that map polarity is lost when spectral amplitudes are computed).

### Microstate Spectral Correlates Between Subjects

In analogy to the within subject analysis, the individual mean microstate features were used as regressors on the individual mean spectral amplitude maps, resulting in a covariance map for each microstate feature and frequency band. The significance of these covariance maps was tested using TANCOVAs (Koenig et al. 2008), a randomization-test that globally tests multichannel EEG data for significant associations with external predictors. When the TANCOVA was significant, the covariance map was displayed. To test whether these between-subject covariance maps systematically varied by frequency band, we repeated these TANCOVAs with frequency band as repeated measures factor. Finally and again, the shared variance with the template maps was computed and tabulated.

## Results and Discussion

In the within subject analyses, 114 of 126 obtained covariance maps (90.5%) were found to be consistent among subjects in the TCT (p < 0.05, Fig. 1), most of them with p-values that would survive even hard Bonferroni corrections for multiple testing. This further undermines an early, and meanwhile repeatedly questioned claim that these two EEG measures are mostly unrelated (Lehmann et al. 1993; Koenig et al. 2002; Britz et al. 2010; Milz et al. 2017; Javed et al. 2019; Croce et al. 2020). Consistent covariance maps were found for all frequency bands. In addition, the TANOVAs computed on these covariance maps yielded highly significant interactions (< 0.0001) for all three features, indicating that their topographies depended systematically both on the frequency band and the microstate class. For microstate duration and occurrence, the within subject covariance maps often followed a pattern that replicated the extrema of the template maps: at scalp locations close to the template maps’ maximum or minimum, EEG spectral amplitudes increased with the duration of the microstate, and (except for class C) decreased with its number of occurrences. This qualitative observation was further corroborated by the quantification of spatial variance shared between covariance and microstate template maps was that particularly high in the alpha band and for class C, but also high in the theta and beta bands and for other microstate classes (Table 1). Accordingly, across-class microstate duration and occurrence were associated with widespread and broadband increases (for duration) and decreases (for occurrence) of EEG spectral amplitude. Within subject, the increase namely of alpha band activity thus seems to stabilize EEG microstates in time, which converges with earlier made associations between alpha activity and EEG microstates (Milz et al. 2017).

**Table 1:**
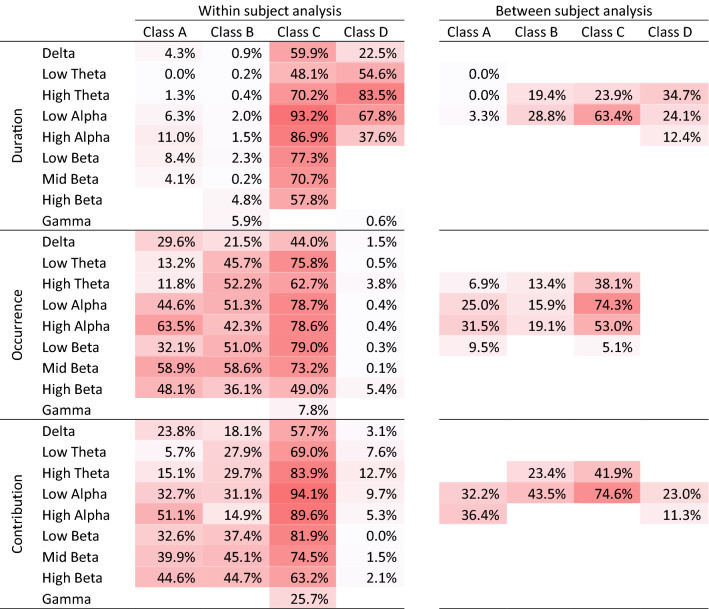
Spatial variance shared among the obtained spectral correlates of EEG microstate features and the corresponding rectified microstate template maps

Cells are color coded by the amount of shared variance for better visualization

Microstate relative contribution, which integrates duration and occurrence, also yielded covariance maps largely driven by the positions of the extrema of the template maps (see Table 1 for shared variance), but spectral amplitude changes were more varied: Spectral amplitude increased with contribution of microstate class C and decreased with microstate class D; for microstate classes A and B, increases were observed near the extrema of the template maps, whereas decreases were seen near the zero-line of the template maps. This confirms the theorical proposal that EEG microstates may exhibit up- and downregulatory control over the excitability of local brain networks (Michel and Koenig 2018). The particularly strong association of EEG microstate contribution with alpha-oscillations also dove-tails with the view that these oscillations represent possible gating mechanisms that improve task performance by inhibiting irrelevant brain regions (Jensen and Mazaheri 2010). EEG microstates therefore present themselves as theoretically interesting, and timewise excellently resolved brain state markers.

In the between-subject analysis, the TANCOVAs were significant only in theta and alpha frequency ranges (32 of 126 covariance maps) and captured mostly the variance of few occipital electrodes. This may in part explain why spectral correlates of EEG microstates tended to be overlooked. In addition, and in contrast to the within subject analyses, between-subject covariance maps were quite similar across microstate classes and frequency bands. Indeed, using frequency band as repeated measures factor in the TANCOVAs did not yield a significant microstate feature × frequency band for any microstate feature. The obtained covariance maps resembled the typical distribution of eyes-closed alpha-range spectral amplitude maps, indicating that subjects with longer and thus less microstates per time had more of the typical occipital alpha activity. When looking at microstate contribution, the average percent time spent in microstates of class A and D correlated positively with this alpha activity, whereas the opposite was found for microstate classes B and C, corroborating earlier conclusions that microstate classes C and D may have antagonistic roles in regulating brain functions (Rieger et al. 2016; Croce et al. 2020).

To conclude, this brief communication points at an interesting avenue for future research in EEG, but it also has a series of shortcomings. On one side, it would be worthy to expand the employed methodology to larger samples with higher spatial sampling and recorded in different conditions. On the other side, an obvious next step is to repeat this type of analysis using frequency domain inverse solutions, and timewise better resolved methods for the assessment of the spectral variance (Javed et al. 2019). Finally, the purely correlative approach employed here leaves open space for systematic research on the causal structure of the interdependencies among different EEG frequencies and EEG microstates.

## Data Availability

The datasets analysed during the current study are available from the corresponding author on reasonable request.
